# The anatomical variant of high soleus muscle may predispose to tendinopathy: a preliminary MR study

**DOI:** 10.1007/s00276-021-02768-9

**Published:** 2021-05-25

**Authors:** Eleni E. Drakonaki, Khaldun Ghali Gataa, Pawel Szaro

**Affiliations:** 1 Musculoskeletal Radiology Practice, Heraklion Crete, Greece; 2 Department of Radiology, Institute of Clinical Sciences, University of Gothenburg, Göteborgsvägen 31, 431 80 Gothenburg, Sweden; 3 Department of Musculoskeletal Radiology, Sahlgrenska University Hospital, Gothenburg, Sweden; 4 Department of Descriptive and Clinical Anatomy, Medical University of Warsaw, Warsaw, Poland

**Keywords:** Achilles tendon, Tendinopathy, Leg, Soleus muscle, Variation, Anatomy, Regional

## Abstract

**Purpose:**

This study aimed to examine the anatomic variations at the level of the distal soleus musculotendinous junction and the possible association between the length of the free tendon and the development of symptomatic Achilles tendinopathy.

**Methods:**

We retrospectively assessed 72 ankle MRI studies with findings of Achilles tendinopathy (study group, 26 females/46 males, mean age 52.6 ± 10.5 years, 30 right/42 left) and 72 ankle MRI studies with normal Achilles tendon (control group, 32 females/40 males, mean age 35.7 ± 13.7 years, 42 right/30 left side). We measured the distance from the lowest outline of the soleus myotendinous junction to the proximal outline of the Achilles tendon insertion (length of the free tendon, diameter a) and to the distal outline of the insertion (distance B). We also measured the maximum thickness of the free tendon (diameter c) and the distance between the levels of maximum thickness to the proximal outline of the Achilles tendon insertion (distance D). All measurements were assessed twice. Statistical analysis was performed using independent *t* test.

**Results:**

Distances A and B were significantly larger in tendinopathic tendons (59.7 and 83.4 mm, respectively) than normal Achilles tendons (38.5 and 60.8 mm, respectively) (*p* = 0.001). Mean distance C was larger in tendinopathic than normal tendons (11.2 versus 4.9 mm). Distances C and D were significantly larger in males than females. There was no significant difference in the measurements between sides.

**Conclusion:**

There is wide anatomical variation in the length of the free Achilles tendon. Tendinopathy may be associated with the thicker free part of the Achilles tendon. The anatomical variant of the high soleus musculotendinous junction resulting in a longer free Achilles tendon may be a predisposing factor to the development of tendinopathy.

## Introduction

The Achilles tendon is the largest tendon in the body, formed by twisted subtendons from the triceps surae muscle complex [21, 26, 29]. The subtendon from the medial head of the gastrocnemius forms the posterior layer, the subtendon from the gastrocnemius's lateral head contributes to the anterior part while the soleus subtendon composes the central and medial part of the Achilles tendon [21, 30]. Based on MR and anatomical studies, the tendon is divided into anatomical segments, including the intramuscular part, the free tendinous region, and most distally, the calcaneal part. The free tendinous region is further divided into the proximal, middle, and distal third [26, 27, 32]. The tendon has a complicated variable blood supply with poor vascularization at the tendon midsection, which is thought to influence the development of tendinopathy [32].

Although the Achilles tendon’s internal structure and vasculature have been extensively studied, many anatomical aspects regarding development of the hypoxemic tendinopathy remain unaddressed [21, 22]. In the last decades, higher occurrence of Achilles tendinopathy in both recreational and professional athletes is documented. Thus, there is substantial demand for detailed knowledge of the Achilles tendon anatomy and variation, which allows a more profound understanding of the biomechanics and risk factors of tendinopathy [10]. Modern magnetic resonance imaging (MRI) has given new insights into the anatomy and morphology of the Achilles tendon and its surrounding structures and has allowed the accurate diagnosis of tendinopathy, based on signal and morphological tendon changes [7, 23, 24, 26]. MRI is nowadays a highly accurate tool to research musculoskeletal anatomy [23] in the living, as it provides anatomic data highly comparable to traditional surgical or cadaveric techniques [24].

During routine MR imaging examinations of the Achilles tendon in the clinical practice, we have noticed a considerable anatomical variation in the level of the soleus muscle musculotendinous junction and the length of the free tendon and the calcaneal insertion. However, there is scarce literature addressing those anatomical variations and their possible role in pathology development [2].

This retrospective clinical imaging anatomy study aims to (1) describe the anatomic variation of the location of the myotendinous junction of the soleus muscle in relation to the proximal outline of the Achilles bony insertion by measuring the length of the free Achilles tendon (2) to investigate the possible association of the length of the free Achilles tendon and the calcaneal insertion to the development of symptomatic Achilles tendinopathy and (3) to express the level of maximum tendon thickness in both normal and tendinopathic Achilles tendons in relation to the free tendon length and the distance from the proximal calcaneal insertion.

## Materials and methods

Study group: We retrospectively reviewed 144 ankle MRI studies (72 right/72 left) of 86 men (mean age 44.4 ± 13.6, range 18–72 years) and 58 women (mean age 43.7 + 16.5, age range 18–69 years) performed between Jan 2018 and Jan 2020 as part of the clinical routine (Table 1). For all patients included in the study, the clinical indication for imaging was available. The study group (group 1) included 72 MRI studies of patients who were referred for imaging with clinically suspected and MRI confirmed Achilles tendinopathy (group 1, comprising 26 females/46 males, mean age 52.6 ± 10.5 years, range 29–72 years, 30 right/42 left side). The control group (group 2) included 72 MRI studies with clinical indications and MRI findings that were not related to the Achilles tendon (group 2, comprising 32 females/40 males, mean age mean 35.7 ± 13.7 years, range 18–69 years, 42 right/30 left side) (Table 1).

**Table 1:** Demographics of the study group (group 1, tendinopathy) and control group (group 2, normal tendons)

	Age (years) mean ± SD (range)	Sex (female/male)	Side (right/left)
All patients	44.1 ± 14.8 (18–72)	58/86	72/72
Group 1	52.6 ± 10.5 (29–72)	26/46	30/42
Group 2	35.7 ± 13.7 (18–69)	32/40	42/30

*SD* standard deviation

MRI protocol, inclusion and exclusion criteria. The MRI examinations were performed using dedicated ankle coils on different MR scanners with field strengths of either 1.5 or 3.0 T and different examination protocols; however, in all cases included in the study, T1-w (weighted), T2-w, and PD-w fat sat (saturation) or STIR (short tau inversion recovery) sequences were available in the axial and sagittal planes. The most common protocol in a 3 T machine (Ingenia Philips) was the following: PD-w TSE (turbo spin echo) TE (the echo time) 45 ms, TR (the repetition time) 2800–5000 ms. T2-w (TSE) TE 60 ms, TR 3000–5000 ms. T1-w: TE 11.5 ms, TR 700–750 ms. Voxel 0.45 × 0.53 × 3.0 mm, slice thickness 3 mm, FOV (field of view) 14 cm. In all cases, a dedicated ankle coil was used and at least the following sequences were available: PD-w or T2-w or T1-w sequences with or without fat saturation in the sagittal and axial planes.

Clinical diagnosis of tendinopathy was provided in the referral letter. The MR diagnosis of Achilles tendinopathy was based on the presence of an abnormally high intra-tendinous signal in T2-w (T2-weighted)/PD-w (proton density weighted) fat sat axial and/or sagittal sequences, which may coexist with maximum tendon thickness of more than 6 mm and convex anterior margin of the tendon in axial images [7, 23, 24]. Small punctate areas of the high signal at the distal Achilles tendon on T2-w in axial images were not considered a sign of tendinopathy [24].

Exclusion criteria were Achilles tendon rupture, history of previous calcaneal fracture (two cases), and artifacts due to orthopedic hardware or motion (six cases). All examinations were reviewed by a musculoskeletal radiologist and a radiology resident and the final results were reported by consensus.

Anatomical MRI measurements: All measurements were performed using freehand electronic calipers on the PACS workstation platform. All measurements were performed twice, and the mean value was recorded. Specifically, the following measurements were performed.

Distance A: The free Achilles tendon length, defined as the distance between the most distal point of the soleus muscle fibers insertion (myotendinous junction) to the anterior aspect of the Achilles tendon, to the superior calcaneal insertion of the Achilles tendon. The localization of the most distal point of the soleus muscle fibers was first identified on axial images and then localized on the sagittal images to measure the length.

Distance B: The distance between the most distal point of the soleus muscle fibers insertion to the anterior aspect of the Achilles tendon (localized as described previously), and the most distal calcaneal insertion of the Achilles tendon. The measurement was performed in the sagittal plane.

Distance C: The maximum thickness of the free tendon, defined as the anterior–posterior dimension of the thickest part of the Achilles tendon. The measurement was performed in the axial plane and confirmed in the mid-sagittal plane.

Distance D: The distance from the maximum tendon thickness level (as described for distance C) to the Achilles tendon’s proximal calcanean insertion.

The D/A% ratio: the % ratio of distance D divided by distance A (D/A × 100)%, expressing the relative distance of the maximum tendon thickness level to the proximal calcaneal insertion.

Statistical analysis: statistical analysis was performed using SPSS 21. Differences in mean or median values of the measurements between groups, sexes, and sides were assessed used independent values *t* test and correlation between length and age using Pearson’s correlation. A *p* value of less than 0.05 was considered statistically significant.

The Swedish Ethical Review Authority approved the research protocol, and the need for informed consent was waived (Trial registration: Ref. no DNR 2020-06177/2020-09-12 Swedish Ethical Review Authority).

## Results

**Fig. 1:**
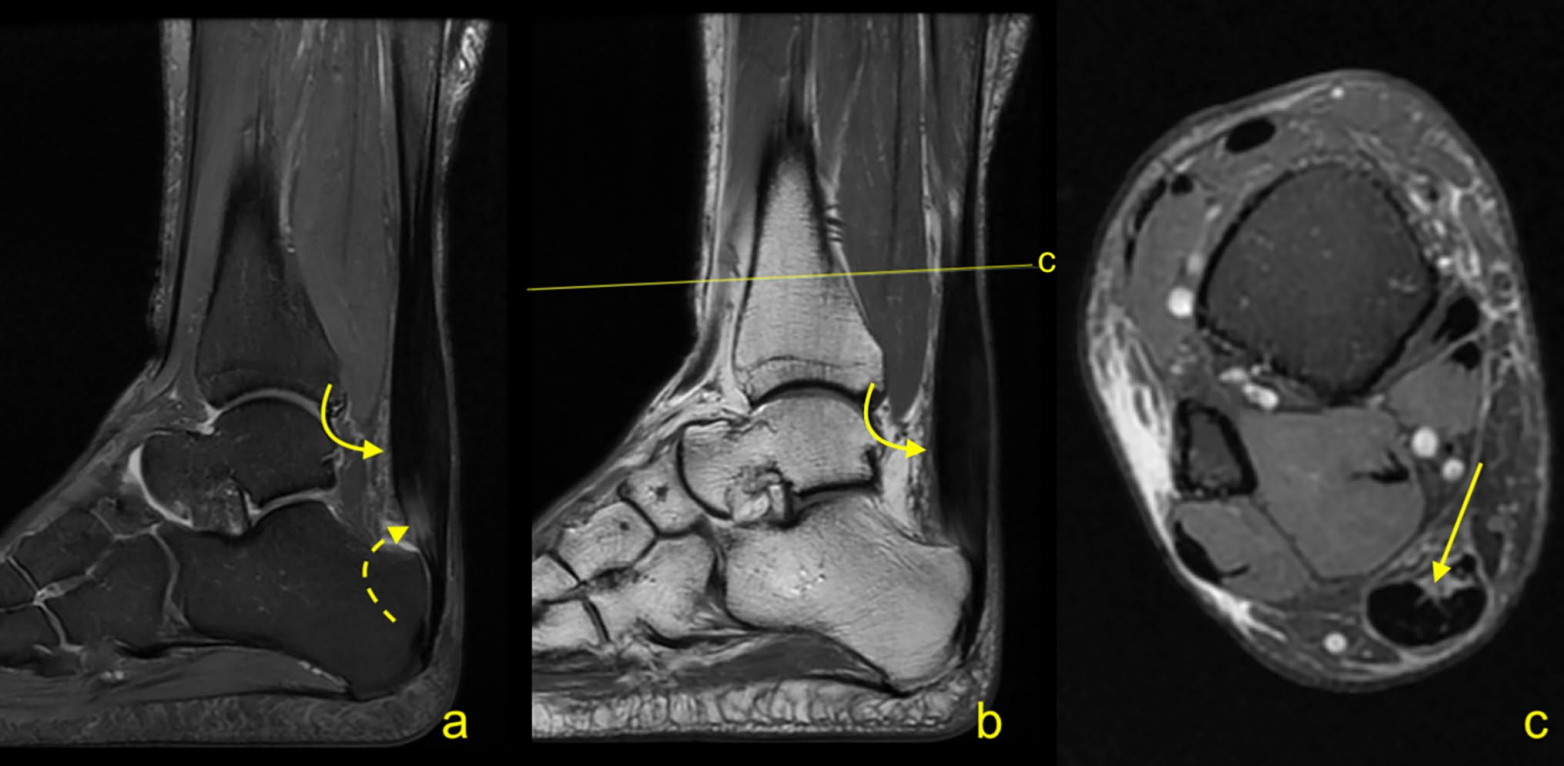
A 34-year-old male with painful Achilles tendinopathy and a long free tendon measuring 77.2 mm. MRI (**a** PD−w fat sat, **b** PD−w, **c** PD−w fat sat) revealed convex outline of the Achilles tendon (curved arrow) with high signal (curved dashed arrow). The lowest axial section (**c**) where the soleus muscle fibers can be identified (arrow)

**Fig. 2:**
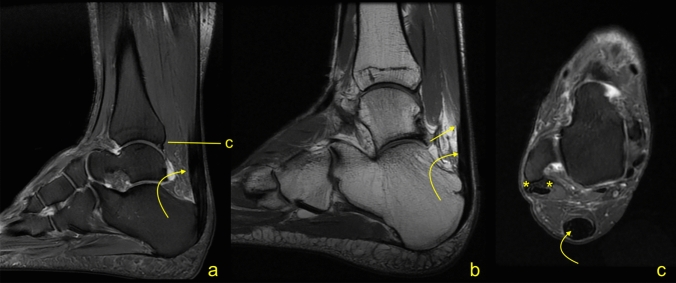
A 45-year-old male with painful Achilles tendinopathy and a short free tendon measuring 28.3 mm. MRI (**a** PD−w fat sat, **b** PD−w, **c** PD−w fat sat) revealed convex outline of the Achilles tendon (curved arrow). MRI revealed split rupture of the peroneus brevis (asterisk on c)

Group 1: the tendinopathic Achilles tendon measurements and the comparison between gender and sides are shown in Tables 1, 2. The study group (Group 1). The mean distance A is 59.7 ± 18.5 mm (range 27.2–106.8 mm), the mean distance B is 83.4 ± 19 mm (range 47–132.6 mm), the mean distance C is 11.2 ± 3.2 mm (range 6–21.2 mm) and the mean distance D is 24.4 ± 4 mm (range 16–32.4 mm) (Table 2, Figs. 1, 2). The mean D/A% ratio is 44.7 ± 17.2% (range 16.6–99.6) (Table 2).

**Table 2:** Differences in the distances (mean ± SD in mm) of the tendinopathic Achilles tendons (group 1) between sexes and sides

Distances	Side			Sex		
	Right	Left	*p* (*t* test)	Male	Female	*p* (*t* test)
A	56.9 ± 19.2	61.8 ± 18.5	> 0.05	59.5 ± 18.6	60.2 ± 19.5	> 0.05
B	81.5 ± 19.5	84.7 ± 19.1	> 0.05	83.3 ± 19	83.4 ± 20	> 0.05
C	10.8 ± 3	11.4 ± 3.4	> 0.05	11.2 ± 3.3	11 ± 3.1	> 0.05
D	24.9 ± 3.9	23.4 ± 4	> 0.05	24.2 ± 4.1	23.6 ± 3.8	> 0.05
D/A% ratio	48.9 ± 18 9	41.7 ± 15.4	> 0.05	45.4 ± 18.3	43.3 ± 15.4	> 0.05

**Fig. 3:**
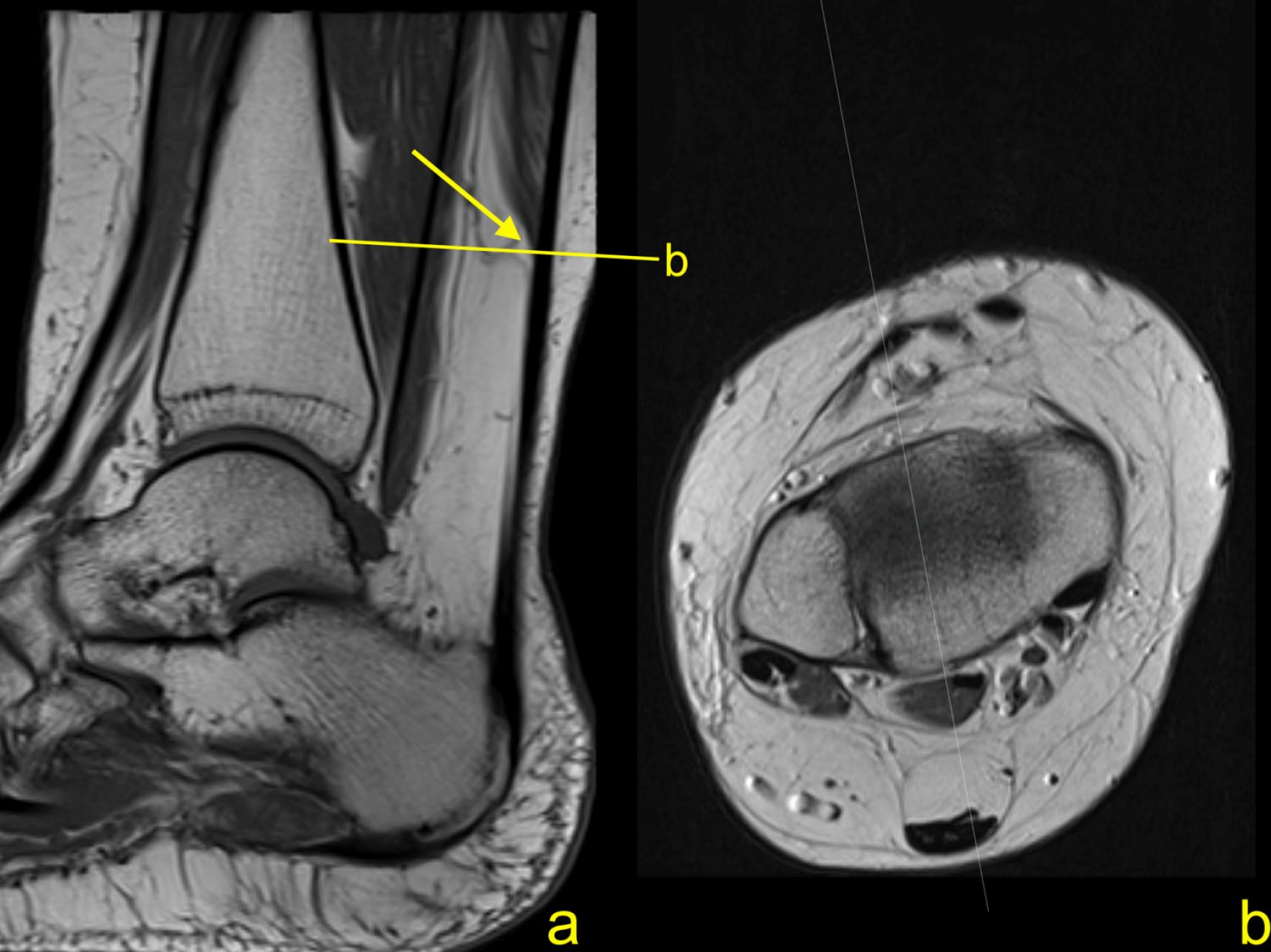
A 39-year-old male with clinical suspicion of the posterior impingement, normal Achilles tendon and a long free tendinous part measuring 71 mm. MRI (**a** T1−w TSE (turbo spin echo), **b** PD−w) showed normal Achilles tendon. The lowest level where the soleus muscle fibers can be identified is marked with an arrow (**a**)

**Fig. 4:**
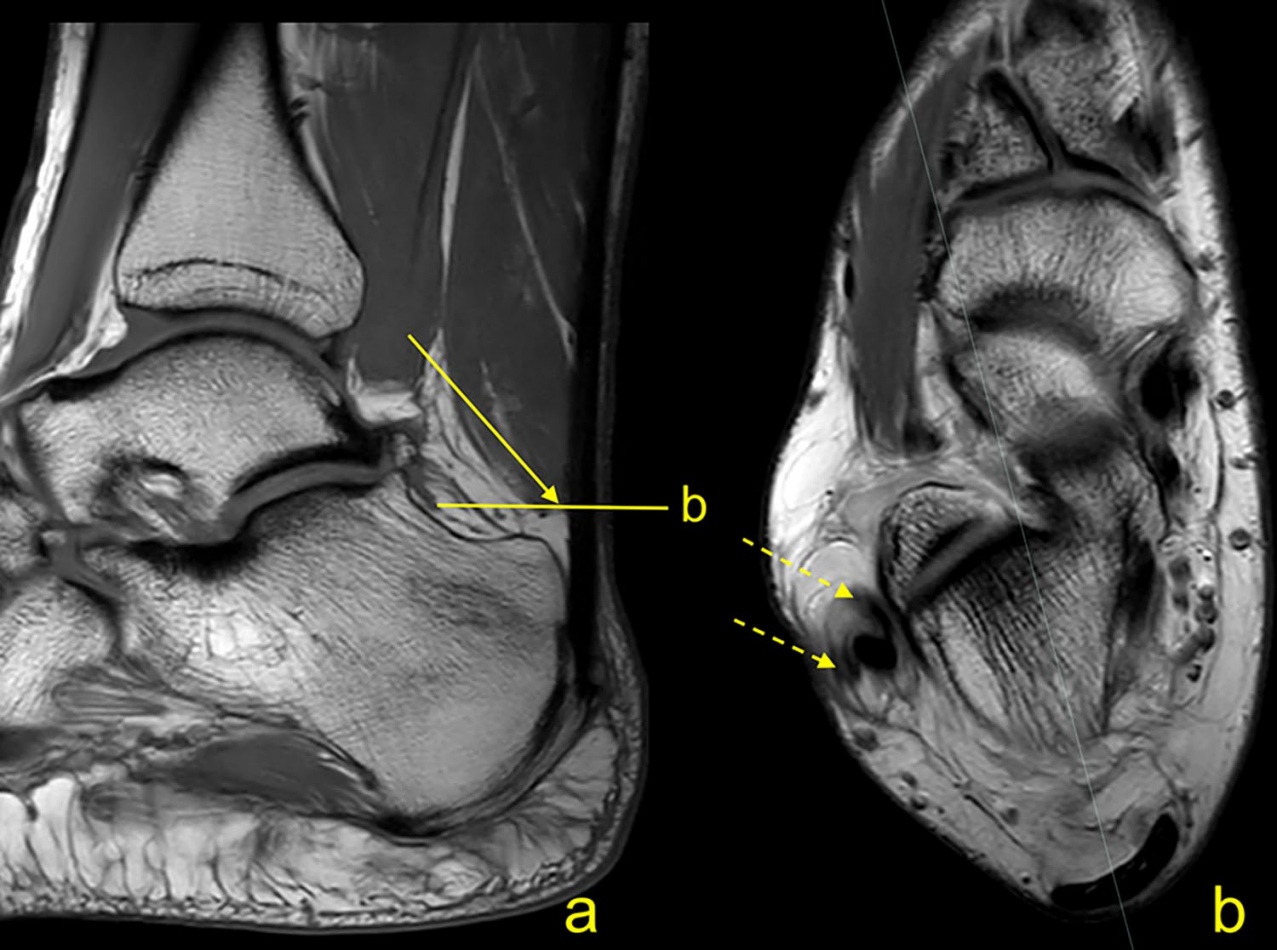
A 31-year-old female with chronic lateral ankle pain, normal Achilles tendon and short free tendinous part measuring 8 mm. MRI (**a** T1–w TSE (turbo spin echo), **b** PD–w TSE) showed the normal Achilles tendon and split rupture of the peroneus brevis as the cause of symptoms (dashed arrows). The lowest level where the soleus muscle fibers can be identified is marked with an arrow (**a**)

No differences in measurements between sex and side were found. The control group (Group 2). The mean distance A is 38.5 ± 14 mm (range 8.5–71 mm), the mean distance B is 60.8 ± 14 mm (range 28.8–97.2 mm), the mean distance C is 4.8 ± 0.4 mm (range 3.9–5.9 mm), and the mean distance D is 18.9 ± 3.7 mm (range 4.2–24.3 mm) (Table 3, Figs. 3, 4). The mean D/A % ratio is 54.1 ± 18% (range 23.2–97.5) (Table 3).

**Table 3:** Differences in the mean distances (mean ± SD in mm) of the normal Achilles tendons (group 2) between sexes and sides

Distances	Side			Sex		
	Right	Left	*p* (*t* test)	Male	Female	*p* (*t* test)
A	37.2 ± 13	40.3 ± 14	> 0.05	38.8 ± 13	38 ± 14	> 0.05
B	59.4 ± 14	62.7 ± 14	> 0.05	61.8 ± 14	59.6 ± 14	> 0.05
C	4.8 ± 0.4	4.9 ± 0.4	> 0.05	5 ± 0.5	4.6 ± 0.3	0.001
D	18.8 ± 3.7	18.9 ± 3.8	> 0.05	19.9 ± 2.5	17.5 ± 4.5	0.006
D/A% ratio	56.3 ± 20	50.8 ± 14	> 0.05	57 ± 19	50.4 ± 15	> 0.05

*Max* maximum, *min* minimum, *SD* standard deviation

The normal Achilles tendon measurements and the comparison between gender and sides are shown in Tables 2, 3. There is no statistically significant difference in the measurements between the right and left sides (Table 2). The maximum thickness of the Achilles tendon (distance C) and the distance of this point to the calcaneal insertion (distance D) is significantly larger in males than females (Table 3). No other differences in measurements between sexes were found.

Comparison between normal and tendinopathic Achilles tendons (groups 1 and 2, respectively).

The comparisons in mean distances between groups 1 and 2 are presented in Table 4. The mean length of the free Achilles tendon (distance A) and the length from distal soleus to distal calcaneal insertion (distance B) are significantly larger in cases with tendinopathy (group 1) compared to normal Achilles tendons (group 2) (59.7 versus 38.5 mm and 83.4 versus 60.8 mm, respectively, *p* = 0.001). The maximum thickness (distance C) of the tendon (distance C) is significantly larger in group 1 than in group 2 (Group 1 11.2 mm versus Group 2 4.9 mm, *p* = 0.001). The distance between the level of maximum tendon thickness, where diameter c was assessed, to the proximal calcaneal insertion (distance D) is significantly larger in group 1 compared to group 2 (24.1 versus 18.9 mm, *p* = 0.001). The D/A% ratio, expressing the relative distance of the maximum thickness area from the proximal calcaneal insertion, is lower in patients with tendinopathy, group 1 (median value 44.7%) than in normal tendons, group 1 (median value 54.1%) (*p* = 0.002).

**Table 4:** Comparison of the a, b, c, d dimensions (in mm) and the D/A% ratio between the tendinopathic (group 1) and normal (group 2) Achilles tendons

	A	B	C	D	D/A% ratio
Group 1 mean ± SD (min–max)	59.7 ± 18.5 (27.2–106.8)	83.4 ± 19 (47–132.6)	11.2 ± 3.2 (6–21.2)	24.4 ± 4 (16–32.4)	44.7 ± 17.2 (16.6–99.6)
Group 2 mean ± SD (min–max)	38.5 ± 14 (8.5–71)	60.8 ± 14.3 (28.8–97.2)	4.9 ± 0.5 (3.9–5.9)	18.9 ± 3.7 (4.2–24.3)	54.1 ± 18.1 (23.2–97.5)
*p* (*t* test)	0.001	0.001	0.001	0.001	0.002

*Max* maximum, *min* minimum, *SD* standard deviation

## Discussion

We found differences among examined individuals regarding the level of soleus distal muscle belly insertion, with the free Achille tendon ranging considerably between 0.85 and 7.1 cm with a mean value of 3.85 cm, with no significant differences between sides and sexes. Only a few papers are assessing the Achilles tendon morphometry in the literature, with a few published studies measuring the length of the free tendon using MRI [1, 2, 12, 25, 27], 3D ultrasound [4, 17, 18], and in cadavers [22]. MRI measurements of the free tendon length reported a mean length of 6 cm ranging between 3.3 and 11.8 cm [2, 27] and a maximum mean length of 7.3 cm [1, 12]. No significant differences between sides were reported [2]. Pilcher et al. [22] measured the free Achilles tendon length in 80 cadavers and found that the mean length was 5.51 cm with 70% specimens ranging between 2.5 and 7.6 cm. Other previously reported values measured using 3D ultrasound ranged from 4.28 ± 1.12 [3], 5.5 ± 1.5 [4], 6 + 1.7 cm [19] to 7.2 ± 2.2 cm [17] for healthy individuals. The present study results are in agreement with the previously reported range of values with a tendency towards shorter tendons in our study group compared to previous studies. The range of values likely reflects differences in study populations in ethnic-epidemiological parameters, exercise level, sex, height, weight, and side dominance or different measurement and analysis methods among various studies.

We also found that patients with tendinopathy had significantly longer free Achilles tendons than patients with normal tendons (59.7 mm versus 38.5 mm, respectively), suggesting that longer free tendons could be more vulnerable to injury. If confirmed by further studies, this finding could significantly impact the clinical practice in sports medicine, as it could be an easily documented risk factor and determine training and rehabilitative decisions. In agreement with our findings, using MRI sagittal images, Weber et al. [31] also found that abnormal Achilles tendons were significantly longer than asymptomatic tendons (83.2 and 45.9 mm, respectively). Devaprakash et al. [3] found that the average free Achilles tendon length of healthy long-distance runners without any injury history was shorter than average reported measurements. This finding could also support the possibility that shorter tendons could provide some advantage for runners. However, in the study by Nuri et al. [17], no difference was found in the free tendon length between patients with tendinopathy and healthy controls.

Although other anatomic parameters, such as the types of the plantaris tendon insertion, have been assessed as a predisposing factor of Achilles tendinopathy [20], limited data exist on the role of the anatomic variability of the soleus muscle and the free Achilles tendon length. The free Achilles tendon is the muscle-free part of the tendon covered by the Kager’s fat. Biomechanical studies have proved that the free Achilles tendon is more vulnerable to mechanical changes and experiences the largest longitudinal strains and mechanical creep during running compared to the muscle-covered tendon part [3, 14, 19]. Therefore, the free tendon may be a more prone to injury. Moreover, it is suggested that dysfunction of the soleus rather than gastrocnemius is the main parameter associated with Achilles tendinopathy, as the deepest part of the tendon mid-portion, where tendinopathy usually occurs, comprises mostly of soleus fibers and undergoes the more significant rotation [30]. Therefore, the level of the soleus musculotendinous junction may be of great biomechanical importance. Kager’s fat is another anatomical parameter considered to have a role in Achilles tendinopathy, as it acts as a shock absorber [16]. The Kager’s fat pad is connected to the anterior aspect of the Achilles paratenon [28]. Thus, it is reasonable to assume that the amount of the Kager’s fat pad in relation to the length of the free Achilles tendon may have biomechanical influence [16]. However, the clinical significance of the wide anatomical variation in the free Achilles tendon length remains mostly unaddressed in the literature.

In our study, the maximum thickness of the normal free Achilles tendon was found to range between 3.9 and 5.9 mm and to be larger in males, in agreement to previous reports also reporting 6 cm as a cut-off point to discriminate a normal from an abnormally enlarged tendon [5, 9, 23, 25, 32]. In a previous MRI study on a population aged 18–70 years old, the normal Achilles tendon’s thickness was 6.1–7.1 mm [11]. In our study, no differences between sides were detected and males had significantly thicker tendons, in agreement to previous studies [23, 25]. The thickest point was located at a mean distance of 1.9 cm from the calcaneus; however, the range was extensive (0.4–2.4 cm from the calcaneus). Interestingly, our study showed that the thickest area is located more proximally in males than in females, a finding that, to our knowledge, has not been previously addressed in the literature. We also found that the tendinopathic Achilles tendons are significantly thicker than the normal tendons. It is well documented in the literature that tendinopathic Achilles tendons are characterized by enlarged volume, cross-sectional area, and thickness due to the pathological alterations in tendon structure [3, 8, 17, 23]. In our patient group, the thickest tendon area corresponding to the tendinopathy area was found at a mean distance of 2.4 cm from the calcaneus ranging between 1.6 and 3.2 cm. In agreement with the literature, our results fall within the range of 2–6 cm above the calcaneus, where tendinopathy and tears usually develop [2, 6, 13, 15]. This area is vulnerable to injury due to poor vascularity and biomechanical factors possibly associated with the soleus muscle–tendon unit [23, 32]. We also found that tendinopathy in our patient group occurred at a more proximal level than the normal thickening of the Achilles of the tendon. However, it could be possible that this finding is due to the significant longer tendons affected by tendinopathy in our study. To eliminate this bias, we suggested using a ratio instead of an absolute measurement to express the relative distance of the thickest area from the calcaneus as a % of the free tendon’s total length.

Due to the wide variation in the level of soleus distal insertion, the distance of 2–6 cm from the calcaneus may correspond to different anatomical areas of the triceps surae tendon complex. In our study, in particular, the lowest soleus insertion level was at 0.8 cm and the highest at 10.6 cm from the calcaneus; thus, the 2–6 cm distance reported by the literature may fall either within the free tendon or at a variable level of the muscle–tendon unit. Using the D/A% ratio to express the relative distance of the maximum thickness area from the proximal calcaneal insertion, we found that the thickest area in both normal and abnormal tendons is located within the tendon mid-portion. The D/A% ratio tends to be lower in patients with tendinopathy (median value 44.7%) than in normal tendons (median value 54.1%), thus suggesting that the thickest area in Achilles tendinopathy usually occurs distally to the free tendon middle compared to normal tendon maximum thickness point that lies proximally to the free tendon middle. However, in both groups, the ratio had a wide range of values ranging from very near the calcaneus (17%) to the soleus’ musculotendinous insertion (100%).

## Study limitations

We acknowledge the presence of limitations in the current study. We used pre-existing clinically used MRI protocols, as the study was retrospective. Measurement errors may have been encountered related to slice thickness and acquisition angle. MRI examinations were performed on two different machines 1.5 and 3.0 T. To improve measurement accuracy, a special protocol using thinner sections or 3D sequences could be used in a future prospective study. Not the entire soleus muscle was included in the study. The absence of surgical correlation may also be a limitation of this study, as well as the lack of information about the subjects’ height, weight, and body mass index (BMI).

## Conclusion

The length of the free part of the Achilles tendon in tendinopathy tends to be longer than without tendinopathy. The anatomical variant of high soleus muscle may be a predisposing factor to the development of tendinopathy. The thickest area in the abnormal Achilles tendon is usually located distally to the free tendon mid-portion compared to the normal tendon maximum thickness point that usually lies proximally to the free tendon mid-portion.

## Data Availability

Yes.

## Code availability

Yes.
